# A Digital Health Solution for Child Growth Monitoring at Home: Testing the Accuracy of a Novel “GrowthMonitor” Smartphone Application to Detect Abnormal Height and Body Mass Indices

**DOI:** 10.1016/j.mcpdig.2023.08.001

**Published:** 2023-12

**Authors:** Thilipan Thaventhiran, Joanna Orr, Joan K. Morris, Anne Hsu, Lee Martin, Kate M. Davies, Vincent Harding, Paul Chapple, Leo Dunkel, Helen L. Storr

**Affiliations:** a Centre for Endocrinology, William Harvey Research Institute, Queen Mary University of London, United Kingdom; b Centre for Genomics and Child Health, Blizard Institute, Queen Mary University of London, United Kingdom; c Population Health Research Institute, St George’s University of London, United Kingdom; d Department of Paediatric Endocrinology, The Children’s Hospital at The Royal London, United Kingdom; e Department of Health and Social Care, London South Bank University, United Kingdom; f UCL Digital Experience, Information Services Division, University College London, United Kingdom

## Abstract

**Objective:**

To develop and evaluate a smartphone application that accurately measures height and provides notifications when abnormalities are detected.

**Patients and Methods:**

A total of 145 (75 boys) participants with a mean age ± SD of 8.7±4.5 years (range, 1.0-17.0 years), from the Children’s Hospital at Barts Health Trust, London, United Kingdom, were enrolled in the study. “GrowthMonitor” (UCL Creatives) iPhone application (GMA) measures height using augmented reality. Using population-based (UK-WHO) references, algorithms calculated height SD score (HSDS), distance from target height (THSDS^DEV^), and HSDS change over time (ΔHSDS). Pre-established thresholds discriminated normal/abnormal growth. The GMA and a stadiometer (Harpenden; gold standard) measured standing heights of children at routine clinic visits. A subset of parents used GMA to measure their child’s height at home. Outcome targets were 95% of GMA measurements within ±0.5 SDS of the stadiometer and the correct identification of abnormal HSDS, THSDS^DEV^, and ΔHSDS.

**Results:**

Bland-Altman plots revealed no appreciable bias in differences between paired study team GMA and stadiometer height measurements, with a mean of the differences of 0.11 cm with 95% limits of agreement of −2.21 to 2.42 cm. There was no evidence of greater bias occurring for either shorter/younger children or taller/older children. The 2 methods of measurements were highly correlated (*R*=0.999). GrowthMonitor iPhone application measurements performed by parents in clinic and at home were slightly less accurate. The κ coefficient indicated reliable and consistent agreement of flag alerts for HSDS (κ=0.74) and THSDS^DEV^ (κ=0.88) between 83 paired GMA and stadiometer measurements. GrowthMonitor iPhone application yielded a detection rate of 96% and 97% for HSDS-based and THSDS^DEV^-based red flags, respectively. Forty-two (18 boys) participants had GMA calculated ΔHSDS using an additional height measurement 6-16 months later, and no abnormal flag alerts were triggered for ΔHSDS values.

**Conclusion:**

GrowthMonitor iPhone application provides the potential for parents/carers and health care professionals to capture serial height measurements at home and without specialized equipment. Reliable interpretation and flagging of abnormal measurements indicate the potential of this technology to transform childhood growth monitoring.

Normal growth occurs in healthy, adequately nourished, and emotionally secure children. Height assessments are considered an essential part of standard pediatric clinical care because abnormal single measurements or growth trajectories (slow or rapid growth) can indicate a spectrum of serious underlying problems.^1^^,^^2^

Short stature is one of the commonest causes for concern among parents and accounts for up to half the referrals to specialist pediatric endocrine clinics.^3^ Pathologic causes of growth failure or short stature are extensive but broadly fall into 2 categories: primary growth abnormalities caused by intrinsic defects of the growth plate (skeletal dysplasia, dysmorphic syndromes, chromosomal disorders, and small for gestational age with failure of catch-up growth) and secondary growth abnormalities caused by a wide spectrum of disorders, which adversely affect the growth plates, such as psychosocial problems, endocrine disorders, and long-term conditions. Tall stature may also indicate serious underlying pathology, for example, dysmorphic syndromes/chromosomal disorders, precocious puberty, and other endocrine disorders. Growth monitoring (serial height measurements) of children with early appropriate referral can facilitate prompt diagnosis and enable timely initiation of appropriate interventions to minimize the negative effect of disease, improve quality of life, and allow catch-up to an optimal final adult height.^4^^,^^5^

Wall-mounted stadiometers are the gold standard to measure standing height in the clinical setting, but these can be costly. Uncalibrated or improperly maintained equipment and interobserver differences in measurement technique can lead to inaccurate height data. For height data to be accurate and reproducible, measurements need to be performed regularly by careful and consistent observers using calibrated instruments.^6^

Height assessment in children is performed by comparison with appropriate population references and comparison with their target heights (genetic height potential) and by height velocity (growth rate). The most used screening parameters (alone or in various combinations) are as follows: (1) height SD score (HSDS), (2) distance of HSDS from target HSDS (THSDS^DEV^), and (3) HSDS change over time (ΔHSDS).^7^^,^^8^ Growth monitoring programs should have sufficient sensitivity to detect pathology and sufficient specificity to prevent the referral of completely healthy patients. It is widely accepted that a combination of all 3 parameters, with appropriate cutoffs, give the highest sensitivities and specificities for the identification of growth disorders.^7^ Childhood growth disorders are frequently diagnosed late, and suboptimal growth monitoring practices contribute to these delays. In Finland, population-based computerized screening algorithms and automated flagging of abnormal growth in electronic health records improved the detection rate of growth disorders and was found to be markedly more effective than the traditional systems in primary care.^9^

Malnutrition, which includes obesity and undernutrition, are becoming increasingly prevalent in populations worldwide. They are the leading causes of poor health, morbidity, and premature mortality and are recognized as major public health prorities.^10^^,^^11^ Early identification allows management strategies to be introduced before the onset of complications, but studies consistently show that parents lack accurate perception of their child’s weight status. This is critical because parents who are aware are more likely to take steps to change their child’s unhealthy lifestyles and prevent malnutrition evolving.^12^ Age- and sex-adjusted body mass index (ISO-BMI) can be used to compare an individual’s body mass index (BMI, calculated as the weight in kilograms divided by the height in meters squared) with the internationally accepted definitions of thinness, overweight, and obesity for children and adolescents.^13^^,^^14^ Accurate ISO-BMI calculations require accurate height measurements and provide additional information during growth assessments.

The aim of this study was to develop a bespoke growth monitoring smartphone application and determine its usefulness, capability to measure height accurately and reproducibly, record serial measurements, detect abnormal values, and provide in-application notifications and recommendations for further actions. Accessible digital height monitoring that incorporates growth algorithms may provide a cost-effective screening tool for detecting abnormal growth in the community and improve the identification and timely referral of disordered growth. The integration of ISO-BMI calculated from manually entered weight provides an additional tool to increase parental awareness and identify overweight/obese children earlier.

## Methods

### Study Design and Participants

Study participants were recruited attending pre-arranged new and follow-up outpatient appointments at the Children’s Hospital at Barts Health Trust, London, United Kingdom. Participant’s heights and weights and their parent’s standing heights were measured by a trained health care professional using a calibrated Harpenden stadiometer (Harpenden) and Seca electronic scales (Seca), respectively. Participants with GMA compatible iPhones (6S-13; iPhone operating system [iOS] 13.5 or later), capacity to consent, and the ability to reliably stand for accurate height measurements were included. Participants who were unable stand unaided and lacked the capacity to consent were excluded.

### GrowthMonitor Application

**Figure 1:**
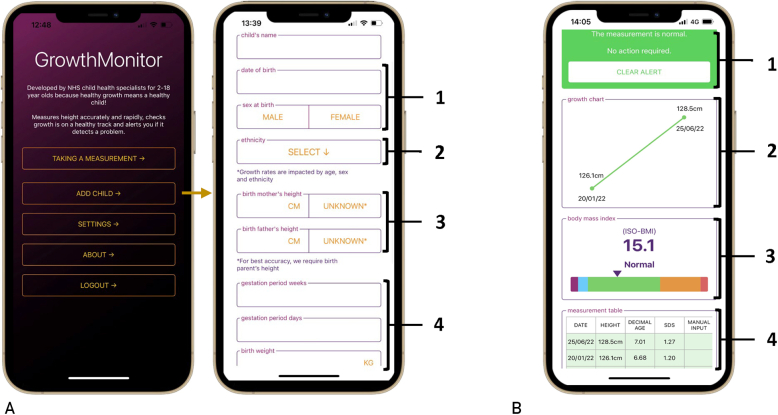
GrowthMonitor application (GMA) input/output interfaces and workflow. (A) The GMA’s functionality is navigated from the home screen. When a profile is created, the user is prompted to manually input demographic data: (1) date of birth and sex to calculate screening parameters in relation to age- and sex-matched reference data; (2) ethnicity for context of height measurement; (3) paternal and/or maternal height to calculate distance from target height (THSDS^DEV^); and (4) gestational age and birthweight to calculate birthweight SDS (BW SDS). (B) Height measurements acquired by GMA are analyzed and compared with pre-established age- and sex-matched UK-WHO reference data. The algorithm to evaluate height SDS requires only the child’s height measurement and is executed first. The second algorithm to evaluate height compared with parent height (THSDS^DEV^) is then executed. The algorithm to evaluate change in height-over-time (ΔHSDS) is executed next. The output is (1) a single flag alert with a recommendation. Other outputs displayed on the scrollable viewport include (2) a growth chart that displays a timeline of recorded heights, (3) a BMI value with color-graded ISO-BMI weight classification if a weight is manually entered following a height measurement, and (4) a table of historical height measurement data with the corresponding decimal age and height SDS. BMI, body mass index; ISO-BMI, age- and sex-adjusted body mass index; SDS, SD score.

The application was developed by an interdisciplinary team of investigators in partnership with expert medical application developers (UCL Health Creatives). GrowthMonitor application uses augmented reality (AR) technology to detect and record standing height measurements. Anonymized height data were automatically assigned unique trial participant identifiers in the application and transferred with individual demographic data entered by the parents/carers or study team (sex, ethnicity, date of birth, birthweight/gestation, current weight, and parents’ heights) to a General Data Protection Regulation-approved secure central UK server. The application user input and output interfaces are shown in Figures 1A and B, respectively. Height measurements were analyzed in the GMA using growth monitoring algorithms.^9^ The GMA algorithms calculate the following: (1) HSDS relative to population-based age-/sex-matched height references (UK-WHO); (2) THSDS^DEV^ using parental heights and population-based height references (UK-WHO); and (3) HSDS change over time (ΔHSDS) if another GMA height measurement was available. Height SD score change over time was only calculated if there was a maximum of 6 months interval for ages 1 year or younger or a maximum of 60 months interval for ages older than 1 year and 12 years or younger between height measurements.

Conditionals and flow control programming enable different types of flag alerts to be displayed when a height measurement is recorded. The algorithm to evaluate HSDS requires the child’s height measurement and is executed first. A single flag alert is displayed in real-time if a screening condition evaluates to true. If the HSDS measurement is normal, the second algorithm to evaluate THSDS^DEV^ is executed. If the conditions for both algorithms evaluate to normal, the algorithm to evaluate ΔHSDS is executed (Supplemental Figure 1, available online at https://www.mcpdigitalhealth.org/). In-application flag alerts were triggered by height measurements (HSDS, THSDS^DEV^, and ΔHSDS) at the same established predefined thresholds: green (normal) at greater than −2.2414 SDS to less than 2.2414 SDS (97.5% of the population), amber alert at less than or equal to −2.2414 SDS or greater than or equal to 2.2414 SDS (≤1.25th or ≥98.75th percentiles), and red (abnormal) alert at less than or equal to 2.8070 SDS or greater than or equal to 2.8070 SDS (≤0.25th or ≥99.75th percentile) (Supplemental Figure 1).

We assessed the ability of GMA to correctly flag measurements (HSDS, THSDS^DEV^, and ΔHSDS), which were greater or less than the aforementioned cutoff values.^15^ We used 3×3 confusion matrices to assess GMA performance in triggering HSDS-based and THSDS^DEV^-based flag alerts against the benchmark set by flag alerts derived from stadiometer height measurements. GMA performance was evaluated by the detection rate (or test sensitivity), defined as the proportion of abnormal measurements with a red flag alert. Serial growth data were displayed in an integrated growth chart, providing the patient with a digital history of their growth over time. Manual entry of the child’s weight after a GMA height measurement enabled display of ISO-BMI with a color scale according to the age- and sex-specific international BMI cutoff criteria, on the basis of the International Obesity Task Force guidelines^14^^,^^16^: severe underweight (thinness grade 3, ISO-BMI ≤16 kg/m^2^; purple), underweight (thinness grade 2, ISO-BMI ≤17 kg/m^2^; blue), normal (ISO-BMI=17-25 kg/m^2^; green), overweight (ISO-BMI >25 kg/m^2^; orange), and very overweight (obesity, BMI ≥30 kg/m^2^; red). Birthweight SDS was calculated using UK-WHO references if birthweight and gestation were entered during a profile set up in the application. An overview of all GMA screens is shown in Supplemental Figure 2 (available online at https://www.mcpdigitalhealth.org/).

### Procedures

Study interventions were conducted in a single routine clinic visit, including obtaining informed consent and measurement of the child’s height and weight by a trained auxologist. Parent’s heights are routinely measured by a trained auxologist as part of the children’s usual clinical care. At the same clinic visit, attending parent(s) heights were measured. A correction of the proprietary and previously published algorithm, which the GMA uses to calculate target height, was used if only 1 parent height was known.^9^ Parents were given instructions on GMA download and setup and a demonstration of the features and functionality. The GMA was used in the clinic by the study team or parents/carers to obtain height measurements using the study iPhone (version 12) or the parent’s iPhones (version 6S-13; iOS 13.5 or later), respectively. A subset of parents/carers also performed 2 or more further height measurements at home using the GMA installed on their personal iPhones. An android GMA is currently under development.

### Questionnaire

The GMA usability was assessed using a survey created on the basis of the usefulness, satisfaction, and ease of use questionnaire^17^ and was constructed using the web-based survey development software SurveyMonkey (Symphony Technology Group; www.surveymonkey.net). Parents/carers responded to 10 questions, each rated on a 5-point Likert scale, ranging from 1 (strongly disagree) to 5 (strongly agree). Reliability testing of the multiple-question Likert scale was performed using Cronbach α measure of internal consistency.

### Outcomes

The primary outcome was the accuracy and precision of the GMA height measurements and corresponding flag alerts in a pilot population compared with the gold standard stadiometer measurements. A secondary outcome was feedback on the GMA usability by parents/carers.

### Ethical Approvals

Ethical approval was granted by the West Midlands Black County Research Ethics Committee (21/WM/0032).

### Statistical Analyses

The GMA was considered sufficiently accurate if 95% of the time the mean height measurements were within ±0.5 SDS of the stadiometer measurements, assuming the mean difference was zero, that is, the application measurement was unbiased. For a probability of 90% to achieve the target confidence interval (CI) width, the sample size needed was 44 pairs of measurements (the estimated sample size for a 1-SD CI). Assuming a dropout rate and incomplete sets of measurements in at least 50% of participants, we proposed an adjusted sample size of 100 and predicted a complete set of 100 paired measurements would detect height measurements ±0.3 SDS from the gold standard measurement. The Bland-Altman method was used to determine the bias between the GMA and stadiometer height measurements, with variation of the results, referred as limits of agreement (LoA), calculated as ±1.96×SD. Linear regression estimated correlations; 3×3 confusion matrices were used to analyze the potential of GMA height measurements to correctly trigger HSDS-based and THSDS^DEV^-based flag alerts against the gold standard data set. The true-positive (TP), true-negative (TN), false-positive (FP), and false-negative (FN) flag alerts were used to calculate accuracy (TP+TN/TP+FP+TN+FN), precision (TP/TP+FP), recall (TP/TP+FN), F1-score (2 × precision × recall/precision + recall), and specificity (TN/FP+TN). Fleiss κ was used to assess the level of agreement of flag alerts between GMA and stadiometer height measurements. Minitab (Minitab LLC, 2021) was used to analyze data. Reporting of the study design and findings is in accordance with the Strengthening the Reporting of Observational Studies in Epidemiology guidelines.^18^

## Results

To assess the performance of the GMA relative to stadiometer measurements in a clinical setting, we recruited 145 (75 boys) eligible patients with mean age ± SD 8.7±4.5 years (range, 1.0-17.0 years), with self-reported ethnicity categorized into the 5+1 classification (31% White, 7% Black, 39% Asian, 5% mixed, 8% other, and 10% not stated). The mean height ±SD of the study cohort was 130.0±27.6 cm (range, 75.1-188.5 cm).

**Figure 2:**
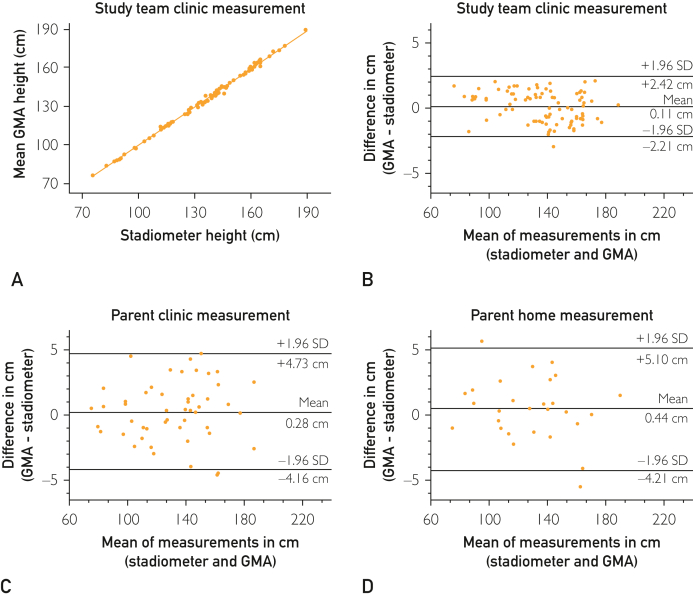
Comparison of the GrowthMonitor application (GMA) and stadiometer height measurements. (A) Height measurements taken in clinic by the research team. Mean of 3 consecutive GMA measurements (cm) vs stadiometer measurement (cm) (gold standard). Linear regression with best fit line, n=88 (Pearson correlation, *R*=0.999). (B-D) Bland-Altman plots. The *x*-axis, average of pairs of height measurements in centimeters; *y*-axis, the difference between the pairs of height measurements in centimeters; green line, the bias (mean difference) between the paired GMA and stadiometer height measurements; red lines, limits of agreement (LoA) estimated as bias±1.96 × SD. (B) Heights recorded using the study team GMA and stadiometer measurement methods in clinic (n=88). (C) Height measurements taken by parents using their own handsets and stadiometer height measurements obtained in clinic (n=50). (D) Height measurements taken by parents using their own handsets at home and stadiometer height measurements obtained in clinic (n=28).

Eighty-eight (46 boys) patients with mean age ± SD 9.8±4.3 years (range, 1.0-17.0 years) underwent 3 consecutive height measurements in clinic using GMA on the study team’s iPhone. To assess the accuracy of the GMA, the mean of 3 consecutive GMA height measurements were compared with the stadiometer height measurement recorded for each patient at the same clinic visit. Linear regression found a significant correlation between the paired measurements (*R*^*2*^=99.7%; *P*<.0001) (Figure 2A). Bland-Altman plots compared and assessed the agreement between GMA and stadiometer height measurements in centimeters. Figure 2B shows the stadiometer measurements compared with the GMA measurements taken by the study team during the clinic visit. These revealed no appreciable bias in differences between paired GMA and stadiometer height measurements. The mean of the differences was 0.11 cm with 95% LoA of −2.21 to 2.42 cm. There was no evidence of greater inaccuracy for either shorter/younger children or taller/older children. The 2 measurements were highly correlated (*R*=0.999). The average coefficient of variance for the repeat height measurements using the GMA was 0.97%, indicating excellent precision. The GMA height measurements had an average error rate of 0.80% and an average accuracy of 99.2% (95% CI, 99.07-99.34). Forty-eight (23 boys) participants with mean age ± SD 9.4±4.3 years (range, 1.0-17.0 years) underwent height measurements taken in clinic by parents using the GMA installed on their personal mobile devices, which were compared with the stadiometer measurements. Figure 2C demonstrates that the GMA measurements taken by parents during the clinic visit were slightly less accurate than those taken by the study team when compared with the stadiometer measurements. The mean of the differences was 0.28 cm with 95% LoA of −4.73 to 4.16 cm. To assess the performance of GMA by nonexpert users in a nonclinical setting, 28 (19 boys) participants with mean age ± SD 8.8±4.6 years (range, 1.0-17.0 years) underwent height measurements taken by parents in their homes using the GMA installed on their personal mobile devices, and these were compared with the stadiometer height measurements taken in clinic. Figure 2D shows that the GMA measurements tended to be slightly more inaccurate relative to the clinic stadiometer measurements when they were taken by the parents at home. The mean of the differences was 0.44 cm with 95% LoA of −5.10 to 4.21 cm. There was no evidence of greater inaccuracy for either shorter/younger children or taller/older children. The 2 measurements were highly correlated (*R*=0.997). Bland-Altman plots also compared and assessed the agreement between height SDS values derived from GMA and stadiometer measurements (Supplemental Figure 3, available online at https://www.mcpdigitalhealth.org/).

**Figure 3:**
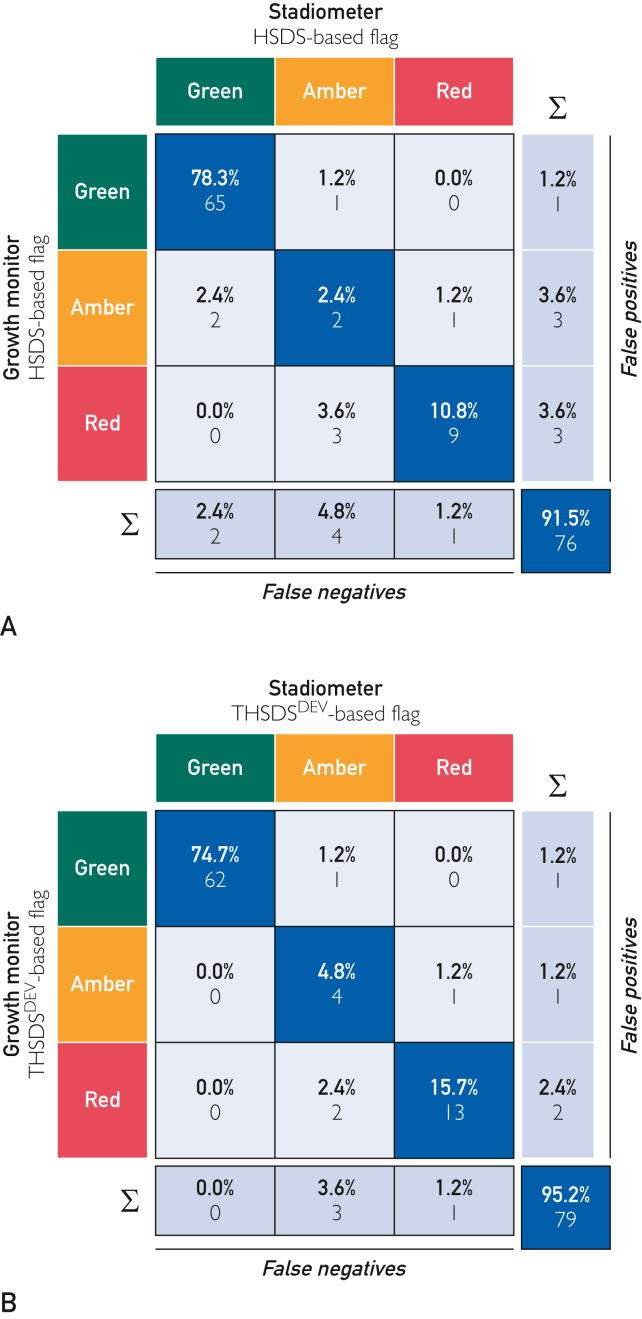
Confusion matrices to evaluate the performance of the GrowthMonitor application (GMA) in triggering the correct flag alerts. The columns represent the true flag alerts generated from algorithm-derived height SDS values obtained from the stadiometer height measurements. The rows represent the flag alert generated from the algorithm-derived height SDS values obtained from GMA height measurements. Each tile contains the normalized count (overall percentage) and the count indicated below. The blue diagonal tiles correspond to the successfully classified GMA flags (true-positive [TP] flags), the light gray tiles correspond to the incorrectly classified flags. The dark gray bottom row expresses the total number of false-negative (FN) GMA flags in each column (true flag). The dark gray column to the right expresses the total number of false-positive GMA flags in each row (predicted flag). True-negative (TN) results for a flag type is the total flag numbers in all columns and rows excluding the flag type in question. (A) HSDS-based flag alerts (n=83). The blue tile at the bottom right-hand side of the plot displays an overall accuracy of 76 of the 83 (91.5%) (HSDS-based GMA flags matched correctly to gold standard). Overall Fleiss κ = 0.74, with an assessment agreement of 91.5% (95% CI, 83.39-96.54). (B) THSDS^DEV^-based flag alerts (n=83). Overall accuracy was 79 of the 83 (95.2%) (THSDS^DEV^-based GMA flag alerts matched correctly with gold standard). Overall Fleiss κ = 0.88, with an assessment agreement of 95.2% (95% CI, 88.12-98.67).

Of the 88 participants, 83 recorded parent height, and 3×3 confusion matrices assessed the performance of the GMA in triggering HSDS-based (Figure 3A) and THSDS^DEV^-based (Figure 3B) flag alerts against the benchmark set by flag alerts derived from stadiometer height measurements (n=83 height measurements). The detection rate for HSDS-based red flags was 96%, with a Fleiss κ value of 0.79 (*P*<.0001), and the detection rate for THSDS^DEV^-based red flags was 97%, with a Fleiss κ value of 0.87 (*P*<.0001). Less normal (green) measurements were detected than would be expected in the normal population. This is likely because most of our patient sample was recruited from a tertiary pediatric endocrine clinic where growth abnormalities are referred, that is, more patients at the extremes of normal measurements.

**Figure 4:**
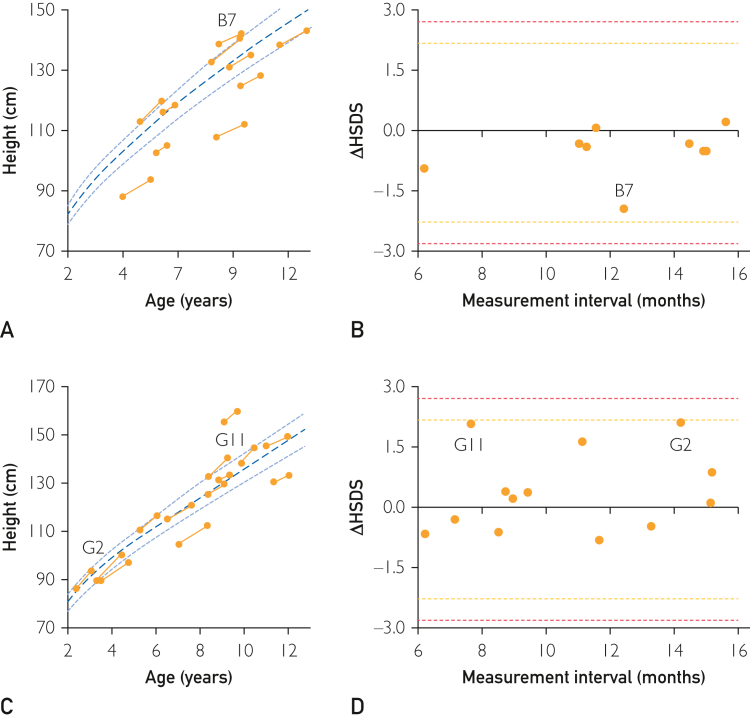
Height change over time from GrowthMonitor application (GMA) measurements. (A) Pairs of GMA height measurements (n=10) plotted against age in years. Dark gray–dashed line, UK-90 population average height for boys (±SD, light gray–dashed line). (B) Algorithm-derived height-over-time (ΔHSDS) values using 2 height measurements 6-16 months apart for boys aged 1-12 years (n=10), compared with UK population–based sex-matched height references. Horizontal-dashed amber and red lines represent the screening cutoffs at ±2.2414 and ±2.8070, respectively. B7 represents the highest ΔHSDS (−2.05). (C) Pairs of height measurements (n=13) obtained from GMA plotted against age in years. (D) Algorithm-derived height-over-time (ΔHSDS) values using 2 height measurements 6-16 months apart for girls aged 1-12 years (n=13), compared with UK population–based sex-matched height references. G2 and G11 represent highest ΔHSDS (2.17 and 2.14, respectively).

Furthermore, 23 (10 boys) participants aged 1-12 years underwent an additional height measurement 6-16 months later, which allowed the GMA to calculate growth rate and use ΔHSDS as a screening parameter. Height SD score change-over-time for boys (Figure 4A) and girls (Figure 4C) were plotted along with the sex-matched UK population average ± SD. Algorithm-derived ΔHSDS from GMA height measurements were plotted for boys (Figure 4B) and girls (Figure 4D) along with the screening cutoff values, as represented by the amber and red-dashed lines in Figure 4. All ΔHSDS values were within the normal range.

Of the 88 participants, 80 recorded their weight. We depicted the distribution of GMA-derived HSDS measurements against the corresponding color-coded ISO-BMI category using a boxplot (Supplementary Figure 4, available online at https://www.mcpdigitalhealth.org/). A significant difference in heights was observed between the severe underweight and very overweight ISO-BMI groups (unpaired *t* test, *P*<.001).

**Figure 5:**
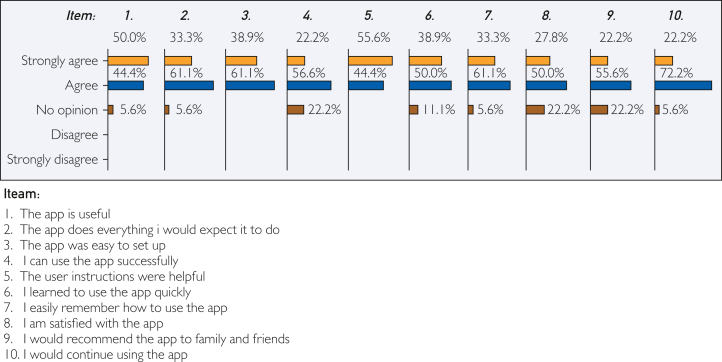
Summary of data from parent and carer questionnaire feedback. Parent and carer feedback (n=18) on GrowthMonitor application (GMA) usability captured from 10 items. Each item was rated on a 5-point Likert scale ranging from 1 (strongly disagree) to 5 (strongly agree). Cronbach α measure of internal consistency gave a value of 0.94, indicating excellent reliability. The mean of the total scale scores for 18 respondents was 42.4±1.11 (of 50), indicating 85% of respondents agreed that the GMA is useful.

To assess the usability of the GMA, 18 parents/carers completed the questionnaire (Figure 5). Cronbach α measure of internal consistency gave a value of 0.94, indicating excellent reliability. The mean of the total scale scores for 18 respondents was 42.4±1.11 (of 50), indicating 85% of respondents agreed that the GMA is useful.

## Discussion

Growth monitoring in children is important because it may detect underlying health conditions in apparently healthy children before other symptoms and signs are present. Frequent height assessment in children with comparison with appropriate population references and target height can reveal anomalous deviations from normal linear growth, which may indicate underlying pathologic growth failure. Growth rate deflection is also a red flag, indicating the need for referral and assessment. This study found the feasibility of a novel smartphone GMA to reliably measure height and provide user-specific recommendations. The performance of GMA was consistent in clinical and nonlinical settings and when used by health care professionals and nonexpert users. Initial usability data were also very encouraging.

The absence of routine growth monitoring can lead to delays in detection and treatment of growth disorders. Self-referrals by activated parents can also lead to unnecessary health care visits and misuse of resources. Currently available growth-related applications rely on the manual input of height or only measure height. The accuracy of current measurement applications varies considerably, and none have been robustly tested and/or have available performance data. The utility of another AR height measurement application was recently reported.^19^ Although this application was found to be user-friendly, the application was only tested in a small cohort of 22 children who were personally known to the investigators, so were likely to have higher motivation than a randomly drawn study sample. Daily measurements were taken over a 3-month period and were not checked for accuracy. Therefore, the suitability for growth monitoring is unclear. The defining feature of the GMA is that the captured height measurements are interpreted by validated algorithms that trigger color-graded in-application notification alerts to confirm normal growth or recommend appropriate medical contact. The GMA remains an application in development, and future interface updates will include integration of a more detailed growth reference chart. Stadiometer measurements are superior in accuracy to those generated by GMA, but as smartphone technology improves, it is predicted that the accuracy of measurement will increase further improving the application’s utility. However, there was a discrepancy between measurements performed by parents in clinic and at home compared with those undertaken by health care professionals. This highlights the need to improve the instructions in the GMA and overall usability.

Our data show that the GMA can measure height with a high level of accuracy and reproducibility. Bland-Altman analysis found a higher level of agreement with stadiometer measurements for GMA height measurements obtained in clinic by the study team compared with parent measurements, which may reflect operator skill and experience. Confusion matrix analysis found a sensitivity (TP rate or correct flag alert) of 91.5% for HSDS-based and 95.2% for THSDS^DEV^-based flags. As a screening parameter in isolation, HSDS has very low sensitivity for growth disorders.^7^ The integration of a second THSDS^DEV^ algorithm allows consideration of the child’s height in the context of their parent(s) height, enabling the identification of children with growth failure in whom height SDS measurements are normal but outside of what would be expected on the basis of genetic target height predictions. Inclusion of this parameter may also avoid unnecessary referrals of short children who have appropriate heights according to their parent(s) heights. Parental height may be unavailable (eg, adoption), inaccurately reported or erroneously estimated. Discrepancies in parent height could limit mid-parental height as a valid adult height predictor. Finally, the accurate calculation of ΔHSDS between the ages of 1 and 12 years will also detect children who have normal HSDS and THSDS^DEV^ but have an abnormal growth pattern. Unfortunately, owing to a lack of time and resources, we were not able to analyze longitudinal data in a bigger sample size. More abnormal (red/amber) measurements were detected in our study than would be expected in the normal population. This is because most of our patient sample was recruited from a tertiary pediatric endocrine clinic where growth abnormalities are referred. Hence, we observe more patients at the extremes of normal values.

Parents frequently underestimate weight status particularly in children at risk of being overweight or overweight.^20^ The GMA calculates a BMI value and displays a color-graded ISO-BMI classification if a weight is manually entered after a height measurement. This is important because parents can better interpret their child’s weight when their BMI is given with an underweight, normal weight, overweight, or obese classification.^21^ The use of colors to represent an increasing risk of abnormality can also improve parents’ perception and empower them to seek medical advice.^22^ Early recognition and intervention in overweight/obese children could prevent problematic complications evolving. For health care professionals, ISO-BMI calculations can also avoid misinterpretation of tall stature in the context of obesity and the identification of poor growth in the context of undernutrition. Obesity during childhood can also alter the dynamics of normal linear growth at different developmental phases.^23^ An upward trend in BMI at ages 2-8 years is associated with increased height gain in early childhood,^24^ reduced height gain in adolescence, and a compromised potential genetic adult height.^25^ In future, the AR-driven imaging technology may be further developed to automatically detect abnormalities in body composition. An additional novel function of GMA is an algorithm-derived birthweight SDS when gestation period and birthweight are entered during profile setup.

Data from the usability questionnaire suggested that parents considered the application useful and easy to use and were satisfied with its function. Limitations included restricted application compatibility to devices that only supported AR technology (iOS device with iOS 11 and an A9 processor or later) and dependence on a stable internet connection to access the cloud-based algorithms before measurements can be recorded. The patients in this study were recruited from a tertiary endocrine clinic and, therefore, did not have a normal height distribution. Hence, the prevalence of screen positive results was high, but this should not affect the estimates of sensitivity. Another limitation of the study included the low response rate for the self-completion questionnaire. These issues could be addressed in future work by integrating feedback surveys into the application with reminder push notifications and undertaking testing in a larger community-based study population. Future implementation work includes development of the application for devices running the Android-operating system.

We report the development, accuracy, and usability of GMA, a novel smartphone application, which can be used to accurately measure child height by health care professionals and parents/carers in a wide range of settings. We found that the novel functionality of GMA to interpret growth measurements using validated growth screening algorithms is effective in triggering in-application notification alerts confirming either normal growth or recommending appropriate medical contact. Hence, the GMA shows potential to be a low-cost, highly scalable, and effective tool to identify disorders of growth in children at any age and prompt timely referral. Age- and sex-adjusted BMI calculation provides an additional tool to increase parental awareness and facilitate the early identification of both malnutrition/underweight and overweight/obesity.

## Potential Competing Interests

The authors report no competing interests.
