# Supplementary material for: A Digital Health Solution for Child Growth Monitoring at Home: Testing the Accuracy of a Novel “GrowthMonitor” Smartphone Application to Detect Abnormal Height and Body Mass Indices
Source: Mayo Clin Proc Digit Health. 2023 Sep 29;1(4):498–509. doi: 10.1016/j.mcpdig.2023.08.001 (PMC10759636; doi:10.1016/j.mcpdig.2023.08.001)

Supplementary Figure S1: Schematic of GMA workflow showing the algorithms and alerts.

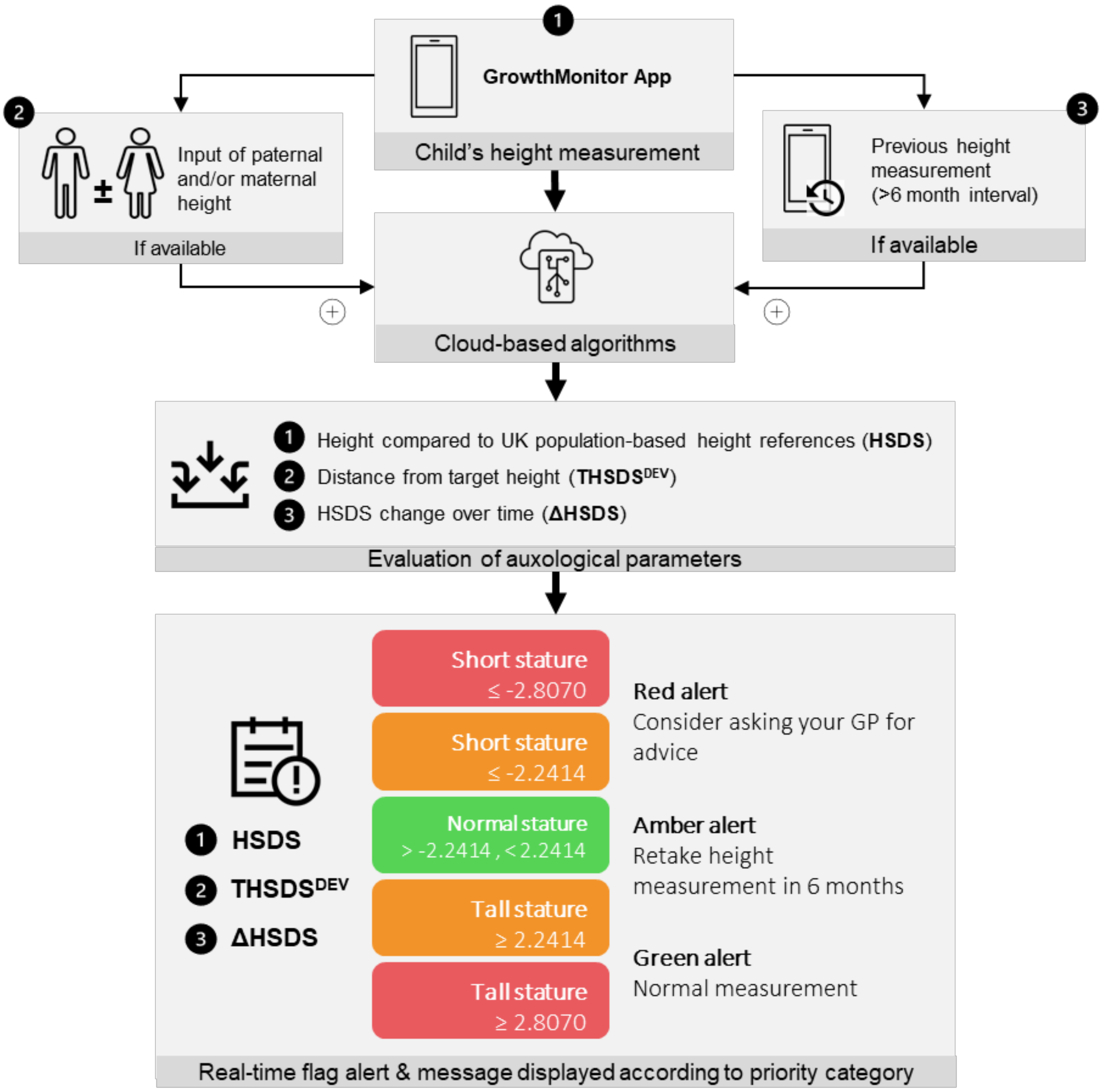

Supplementary Figure S2: 'GrowthMonitor' app (GMA) screen displays

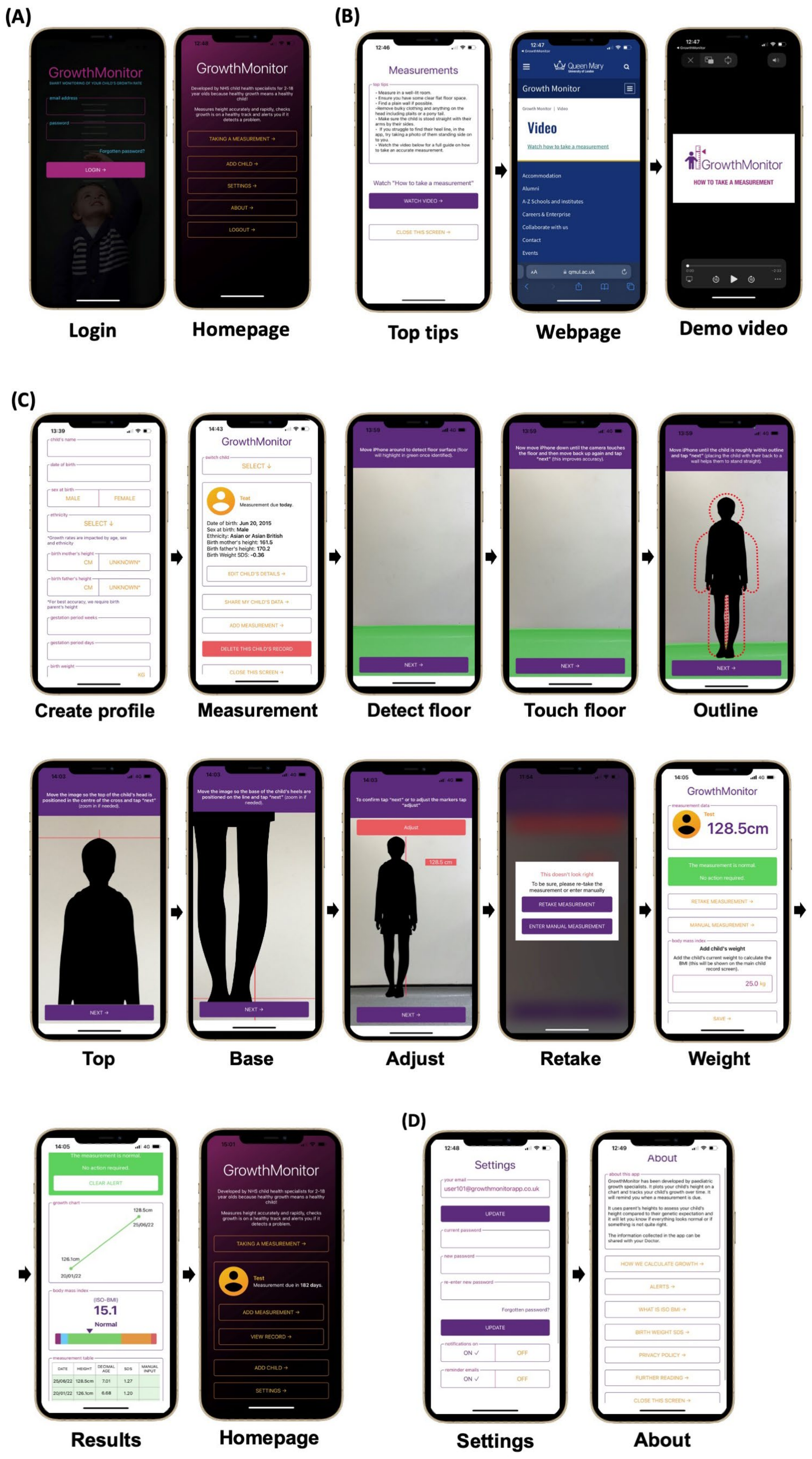

Supplemental Figure S3. Agreement between HSDS derived from GMA and Stadiometer heights.

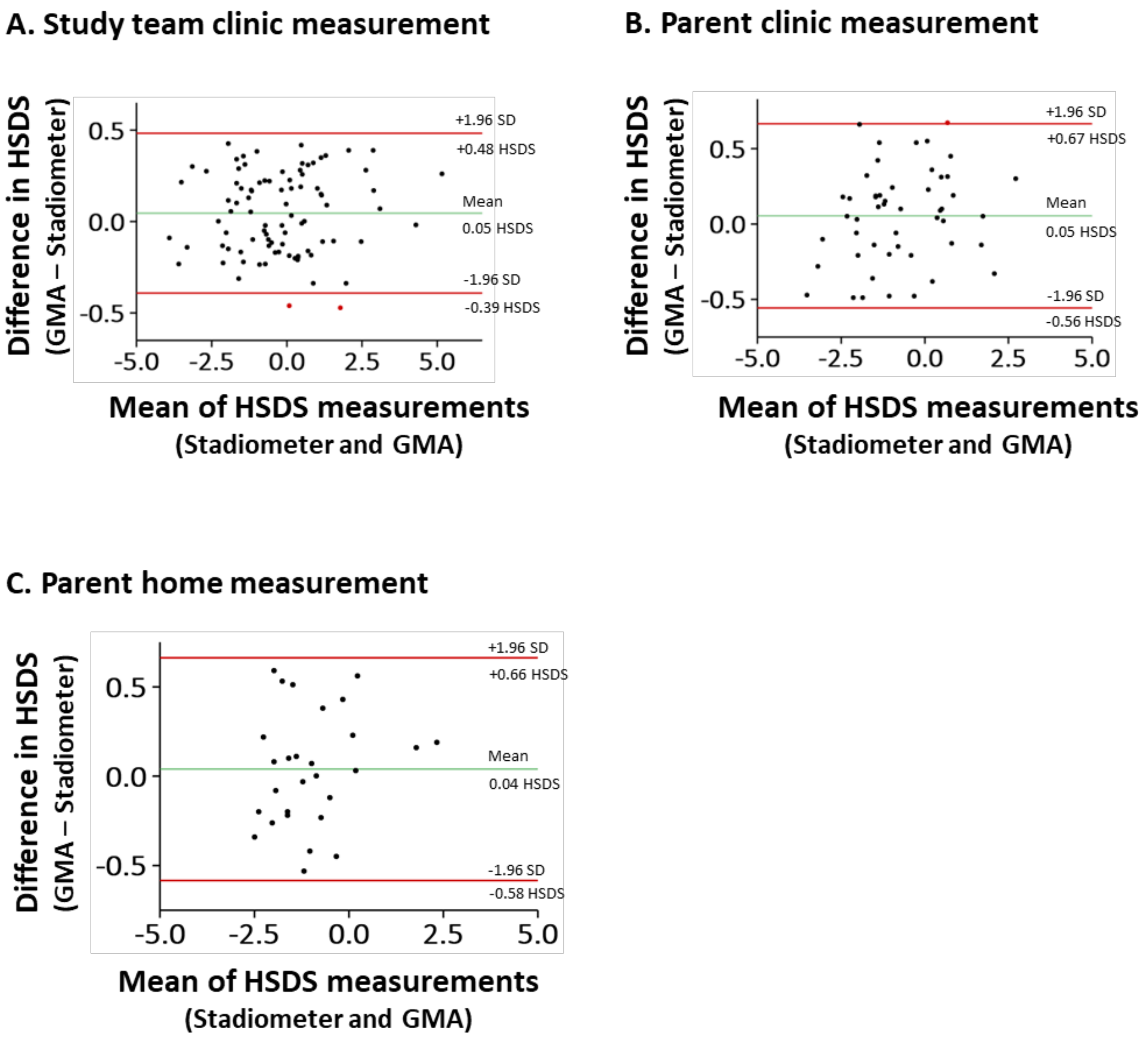

Supplementary Figure S4: 'GrowthMonitor' app (GMA) ISO-BMI data

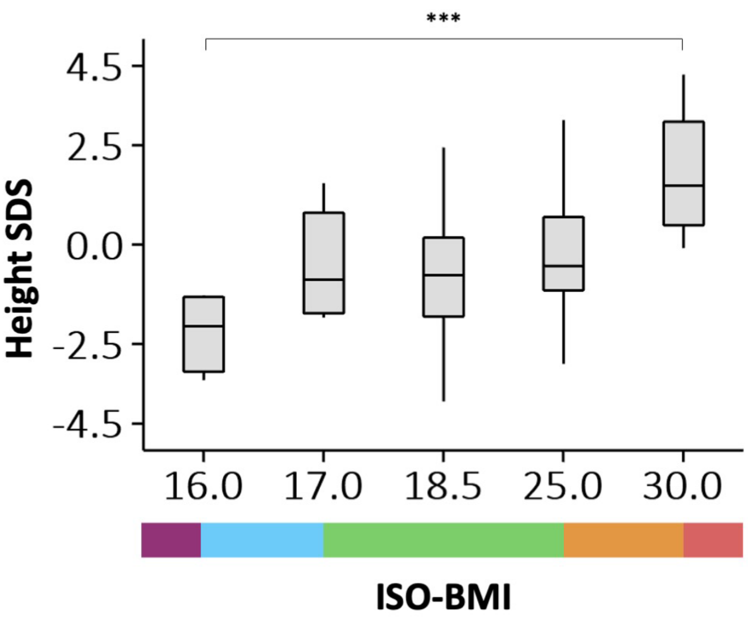

Supplement: Supplementary Material [file mmc1.pdf]
